# Safety and Efficacy of Robotic Hysterectomy Using an Indigenous Robotic System: A Retrospective Study

**DOI:** 10.7759/cureus.81832

**Published:** 2025-04-07

**Authors:** Raj Gajbhiye, Rashmi Solanke, Bhupesh Tirpude, Gayatri Deshpande, Hemant Bhanarkar, Ashutosh Jadhao, Vipin Kursunge

**Affiliations:** 1 General Surgery, Government Medical College Nagpur, Nagpur, IND; 2 Obstetrics and Gynecology, Government Medical College Nagpur, Nagpur, IND

**Keywords:** laparoscopy, robotic hysterectomy, safety and efficacy, ssi mantra, vas score

## Abstract

Background

Minimally invasive hysterectomy approaches, including vaginal, laparoscopic, and robotic hysterectomy, offer advantages over laparotomic hysterectomy. However, vaginal hysterectomy is less feasible for patients with a large uterus, prior pelvic surgery, adnexal surgery requirements, or malignancy, making laparoscopy or robotic surgery preferable. The SSI Mantra robotic system (Sudhir Srivastava Innovations Pvt. Ltd., India) features an ergonomic open-faced console with a 32-inch 3D 4K monitor, a 23-inch 2D touch monitor for system control and DICOM applications, a head-tracking safety feature, and advanced ergonomic controls. This study evaluates the safety and efficacy of robotic hysterectomy using this indigenous system.

Materials and methods

From February to September 2024, 15 patients underwent robotic hysterectomy at Government Medical College, Nagpur. Patients were counseled about using the SSI Mantra robotic system and surgical risks, including possible conversion to laparotomy or robotic arm injury. The procedure followed a standard robotic port approach with accessory laparoscopic ports. Multiple surgeons performed the surgeries. The primary outcome was operative time, defined from incision start to closure. Additional data collected included patient intubation time, draping time, port placement, docking time, console time, surgical end time, patient out time, and estimated blood loss (fluid volume difference between irrigation and suction). Perioperative complications, readmissions, surgeon experience impact on operative time, conversion to laparotomy, hospital stay duration, hemoglobin drop, Visual Analogue Scale (VAS) pain scores, and intraoperative strategies (e.g., adhesiolysis, myomectomy) were analyzed. The weight of the excised uterus was also recorded.

Results

All 15 cases were successfully completed. The mean body mass index was 22.36 kg/m² (range: 19-25). The mean operative time (console use) was 169.41 minutes. Docking time improved from 25 minutes in the first case to five minutes by the 12th. The mean hospital stay was 4.5 days. The average uterine weight was 104.58 g (range: 25-250 g). Estimated blood loss averaged 129.16 mL (range: 30-400 mL). Vaginal cuff closure time was recorded in one case (20 minutes). The mean VAS pain score was 3.91. A hemoglobin drop occurred in four cases, but no postoperative blood transfusions were required. There were no conversions, major complications, or readmissions.

Conclusions

This study demonstrates that the indigenous robotic system provides enhanced ergonomics, seven degrees of freedom, ease of operation, and improved safety with a shorter learning curve for large uteri. Further studies are needed to assess postoperative and long-term outcomes.

## Introduction

Minimally invasive surgical approaches, including vaginal, laparoscopic, and robotic hysterectomy, offer significant advantages over traditional laparotomic hysterectomy, such as reduced postoperative morbidity. However, in cases involving a large uterine size, previous pelvic surgery, the need for adnexal surgery, or malignancy, the feasibility of the vaginal approach is limited, making laparoscopic or robotic surgery the preferred alternative [1].

Traditionally, a midline laparotomy has been used, but it is associated with complications such as organ injury, wound dehiscence, hemorrhage, and infections [2]. Over the past two decades, minimally invasive gynecologic surgery has revolutionized the field, reducing perioperative complications and improving patient outcomes [2]. The American Society of Gynecologic Laparoscopists favors laparoscopic hysterectomy for benign diseases due to its lower postoperative morbidity [3]. However, in cases involving large fibroids or bulky uteri, laparoscopic hysterectomy can be challenging due to difficulties in visualization and manipulation.

Robotic systems, such as the da Vinci surgical system, enhance precision with 360-degree vision and seven degrees of freedom in robotic instruments, facilitating surgery in deep pelvic areas [4]. The da Vinci system, approved by the FDA in 2005, is widely used in gynecological surgeries, including hysterectomy [3]. The SSI Mantra robotic system (Sudhir Srivastava Innovations Pvt. Ltd., India), an indigenous Indian system, offers cost-effective robotic surgery with a 32-inch 3D 4K monitor, modular patient-side arms, and advanced ergonomic features. Its eight degrees of freedom and autonomous camera control improve depth perception and enable complex maneuvers such as suturing, making it an effective option for challenging cases, including large uteri [5].

## Materials and methods

From February 2024 to September 2024, 15 patients underwent robotic hysterectomy at Government Medical College, Nagpur. The Institutional Ethics Committee of the Department of Pharmacology of Government Medical College, Nagpur, issued approval 3641EC/Pharmac/GMC/NGP. All patients were counseled that the SSI Mantra robotic system would be used for gynecologic surgery. Surgical risks, including additional risks such as conversion to laparotomy and injury from robotic arms, were discussed with the patients. The surgery was performed using the standard robotic port approach along with accessory laparoscopic ports. Multiple surgeons performed the hysterectomies. Furthermore, the patients were informed that their surgeons had minimal experience with the SSI Mantra robotic system.

Patients were included in the study if they had large or symptomatic fibroids causing heavy bleeding, pelvic pain, or other complications. Additional inclusion criteria comprised endometrial hyperplasia or malignancy, abnormal uterine bleeding (AUB) unresponsive to medical therapy, and chronic pelvic pain. A history of breast malignancy in the patient or a family history of breast, ovarian, or endometrial malignancy also qualified patients for the study.

Exclusion criteria included morbid obesity (BMI >40), a significantly enlarged uterus (>12-14 weeks size), and severe pelvic adhesions. Patients with severe cardiopulmonary or systemic disease, an inability to tolerate pneumoperitoneum, active infections, or challenges in achieving proper surgical positioning (e.g., due to spinal deformities) were also excluded. Advanced malignancy and vaginal prolapse were additional exclusion factors.

Bilateral oophorectomy may be indicated for several conditions, including ovarian cancer or a high risk of developing it, such as in women with BRCA mutations. It is also recommended in cases of severe endometriosis, benign ovarian tumors or cysts causing complications, and pelvic inflammatory disease (PID) that does not respond to antibiotics. Additionally, this procedure may be necessary for managing hormonal imbalances, such as excessive hormone production, or as a preventive measure for women at high risk of ovarian or breast cancer. It is also considered for severe menstrual dysfunction or chronic ovarian dysfunction.

All the patient records were reviewed, and the demographics and clinical history, including chief complaints, history of present illness, past medical and surgical history, drug history, and perioperative morbidity, were collected. The primary outcome reported included the operative time (defined as the time from the commencement of the incision to closure). Data collection also included parameters such as patient intubation time, draping time, port placement, docking time, surgeon in-console time, surgeon off-console time, surgical procedure end time, patient out time, and estimated blood loss (calculated as the difference in the fluid volume between irrigation and suction). Perioperative complications, readmissions, the relationship between surgeon experience and operative time, conversion to laparotomy, length of hospital stay (defined as the postoperative days elapsed after surgery until discharge), drop in hemoglobin, Visual Analogue Scale (VAS) score, intraoperative strategies like adhesiolysis and myomectomy, and the weight of the excised uterus were studied. Vaginal cuff closure time was also recorded from the procedure.

**Figure 1:**
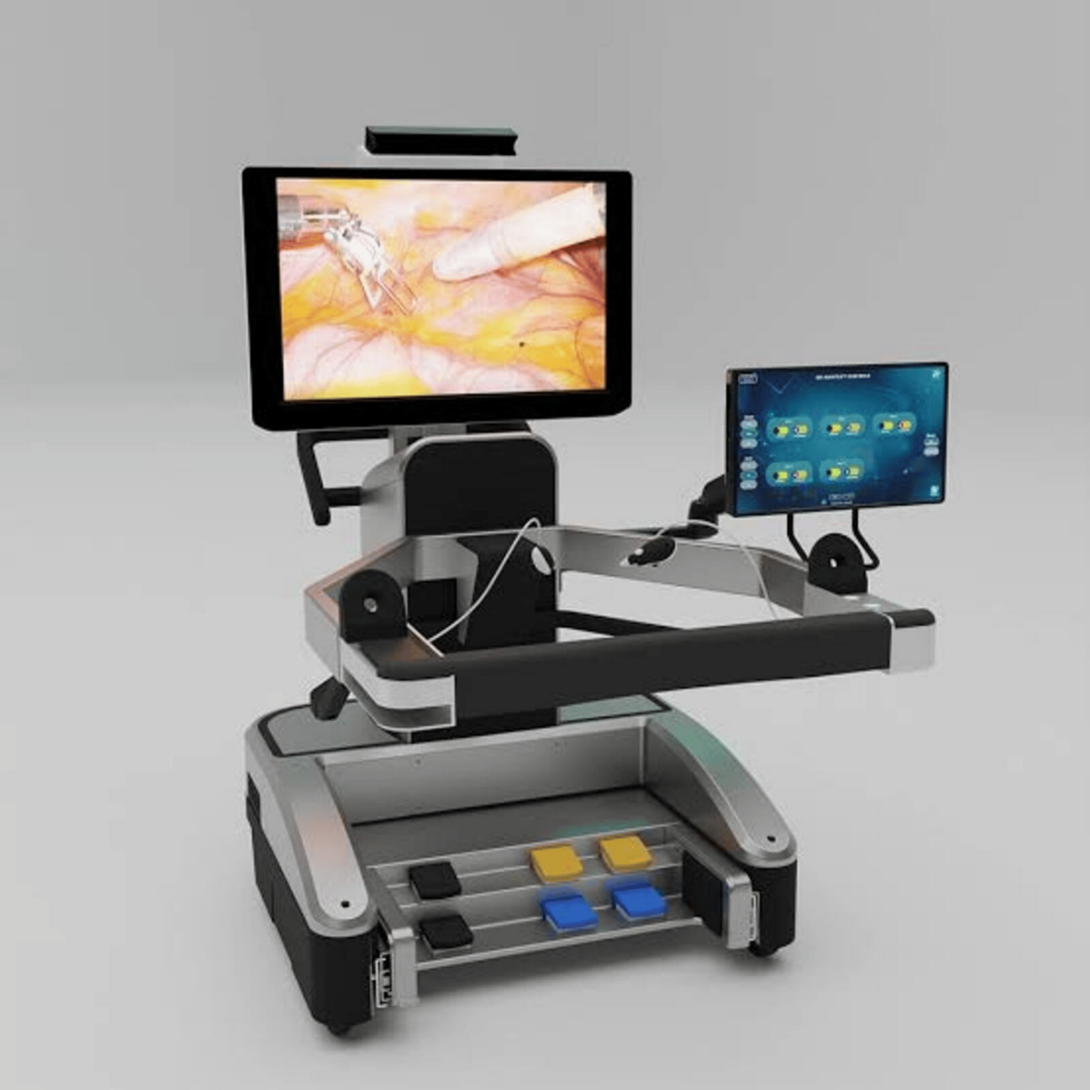
SCC with 3D HD monitor and 2D touch monitor The SCC is an open console featuring a 3D HD monitor and a head-tracking camera. It includes a surgeon hand control device, foot pedal controls at the bottom that house the primary functions of clutch, camera toggle, robotic arm toggle (left and right), cut cautery (left and right), and coagulate cautery (left and right). The SCC also has a 2D touch monitor on the right for turning on the system controls. SCC: surgeon command center, 3D: three dimensional, 2D: two dimensional, HD: high definition Image Credit: Sudhir Srivastava Innovations Pvt. Ltd., with permission

**Figure 2:**
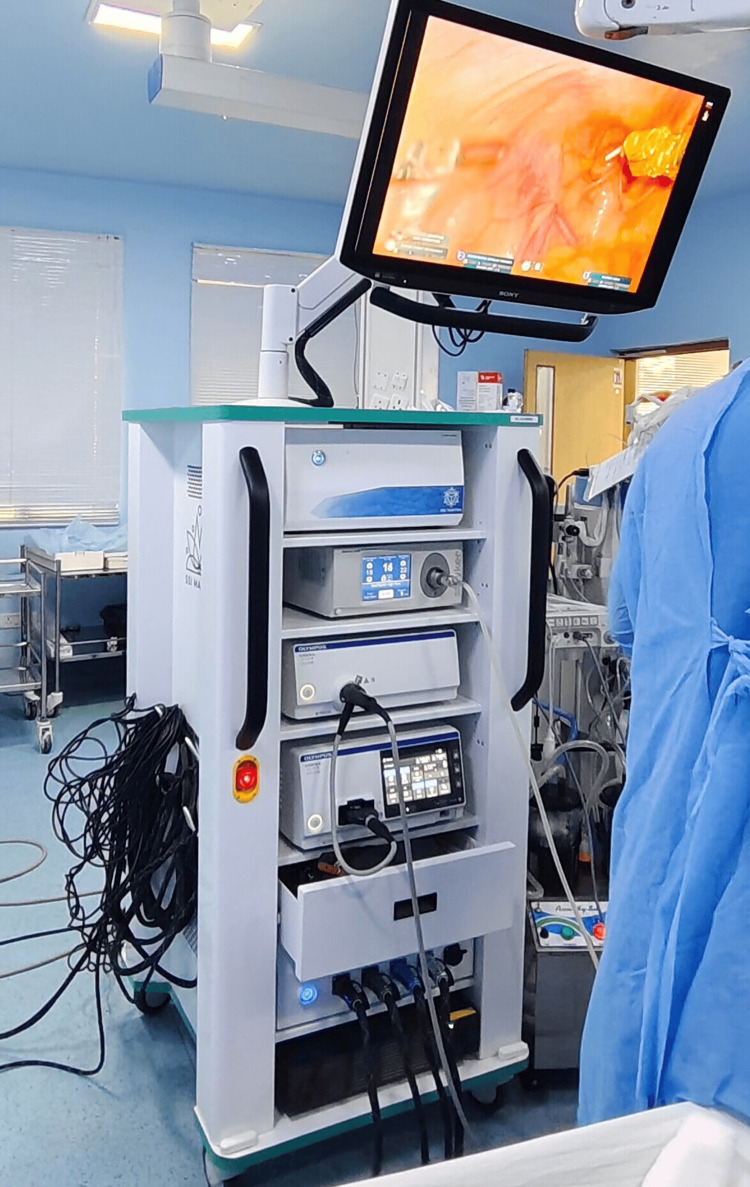
Vision cart The vision cart consists of a 3D HD monitor, a 3D HD recording system, a compartment for the cautery unit, and an endoscope control unit. 3D: three dimensional, HD: high definition Image Credit: Authors

**Figure 3:**
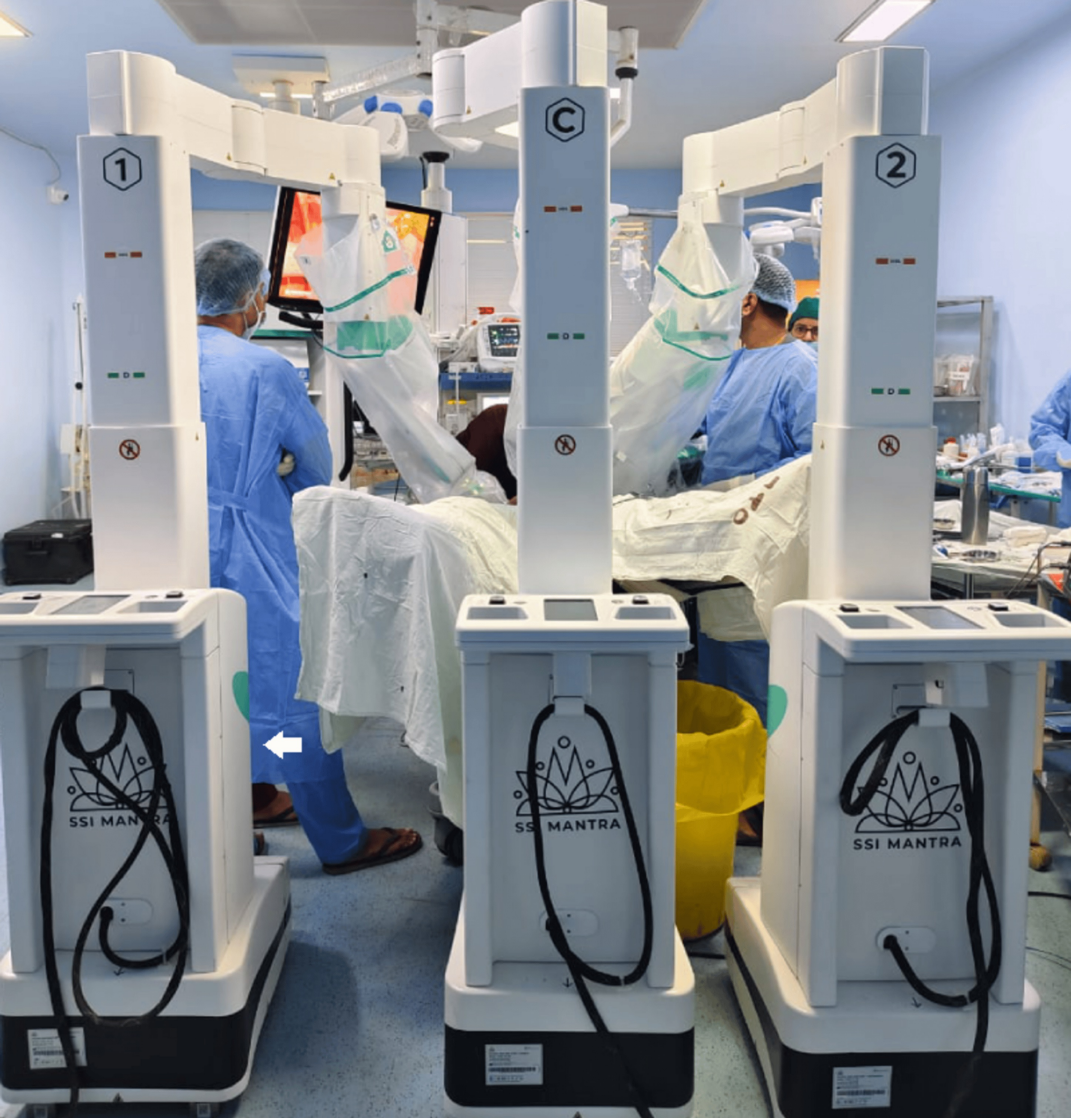
Patient side arm carts The patient side arm carts consist of parking locks, a vertical column, a telescoping column, a cart registration laser, active joints, a tool interface, a camera and instrument actuator, an instrument clutch button, a primary clutch button, and a secondary clutch button. Image Credit: Authors

The indigenous robotic system consists of the open surgeon console (Figure 1), vision cart (Figure 2), patient cart, and robotic arms, including the camera articulating arm. The system has an ergonomic open-faced console, a 32-inch 3D 4K monitor for true depth perception, a large 23-inch 2D touch monitor for system control and DICOM applications, a head-tracking safety feature, and advanced console ergonomic controls (Figure 1). The patient side arm carts (Figure 3) have a modular design, with robotic arms mounted on individual carts and a maximum height of 7.2 feet. The system provides configurations with three, four, or five arms based on the economy and specific surgical requirements. The vision cart features a 360-degree 3D 4K endoscope with a camera length of 375 mm. The 360-degree rotation helps in visualizing the ports without moving the camera. This is one of the most cost-effective robotic systems. The instruments used in gynecologic surgery include the monopolar curved scissors, monopolar hook, Maryland bipolar forceps, fenestrated bipolar forceps, robotic harmonic, needle driver, myoma screw, and Mangeshkar uterine manipulator.

The system displays the surgeon command center (SCC) with a 3D HD monitor to present the endoscopic view of the surgical field in 3D. The surgeon wears trackable 3D glasses to operate the SCC. The surgeon's 3D glasses are tracked by a head-tracking camera located at the top of the 3D HD monitor. With four degrees of freedom, the surgeon uses the hand control device to manipulate the endoscope and instruments. The SCC has a control panel at the front, which houses functions such as power on/off, fault clearing, emergency stop, SCC height adjustment, and foot pedal tray adjustment. The SCC also includes foot pedal controls at the bottom, which manage primary functions such as clutch, camera toggle, and robotic arm toggle.

Figure 2 shows the vision cart positioned outside the sterile field. The primary function of the vision cart is to provide the same 3D HD endoscopic view to the patient-side staff as seen by the surgeon. The vision cart contains the OMNI 3D recording system, space for a cautery unit, an endoscopic control unit, provisions for tools and cables, a system control box, a CO2 cylinder bay, and a preoperative 2D touch panel. Figure 3 shows the patient side arm cart, with side arm carts labeled as C, 1, 2, 3, and 4 for identification purposes. C represents the camera arm, 1 denotes the primary right arm, 2 denotes the primary left arm, 3 denotes the secondary left arm, and 4 denotes the secondary right arm.

Surgeons

Four surgeons and one gynecologist performed the cases. The four surgeons had extensive experience in laparoscopic and oncological surgery, were well-trained in standard robotic port placement, and were naïve to the SSI Mantra robotic system. The bedside team included other surgeons, fellows, and the gynecologist. All the cases were performed by the surgeon and the gynecologist.

Surgical technique

**Figure 4:**
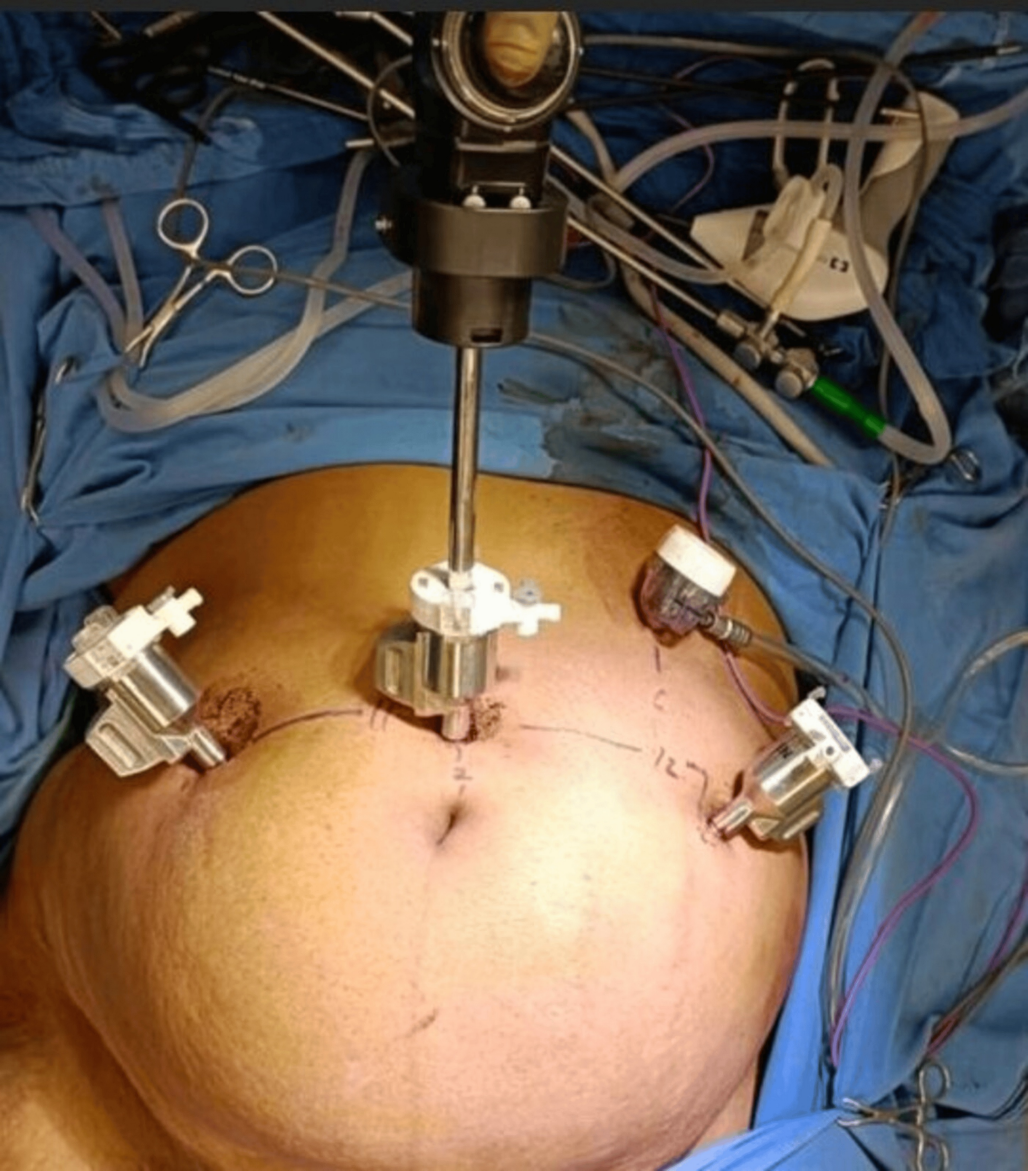
Port placement for hysterectomy The camera port is inserted 2 cm above the umbilicus, and the right and left robotic ports are inserted 5 cm lateral to the camera port on either side. The laparoscopic accessory port is inserted between the camera port and the left robotic port. Image Credit: Authors

Robotic hysterectomy was performed using the SSI Mantra robotic system via four abdominal ports: a 12 mm supraumbilical port, 10 mm right and left ancillary ports, and one 10 mm assistant laparoscopic port (Figure 4). Figure 4 illustrates the port placement, with the camera port (12 mm) positioned 2 cm above the umbilicus. The right and left 10 mm robotic ports were placed 5 cm lateral to the camera port on either side. The laparoscopic accessory port was inserted between the camera port and the left robotic port. Reverse docking was performed to apply the patient cart to the abdominal ports. Monopolar scissors or a monopolar hook were used for dissection, while bipolar fenestrated forceps were employed for vessel sealing. A myoma screw was used for uterine manipulation, or the Mangeshikar uterine manipulator was used by two surgeons.

**Figure 5:**
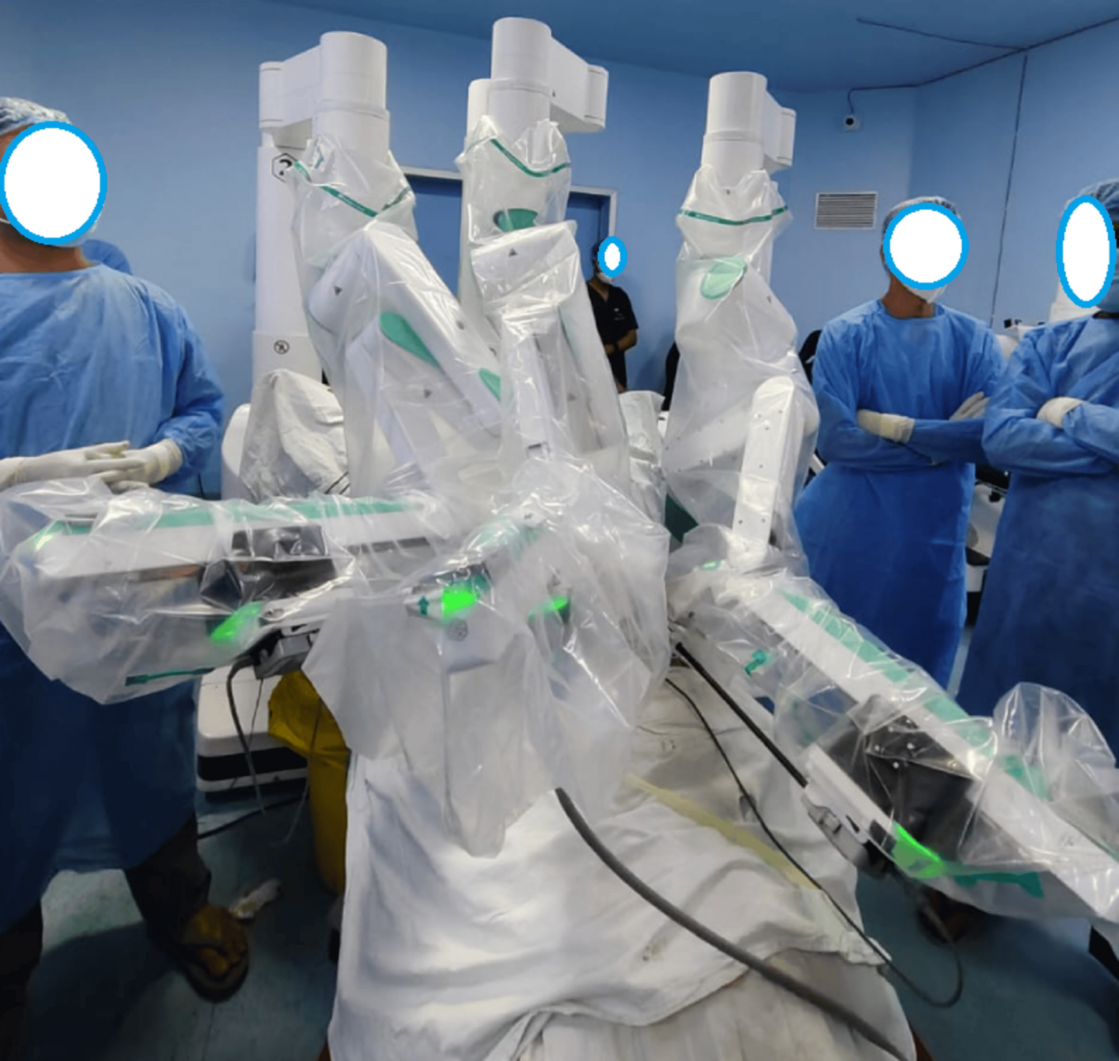
Docked patient side arm cart with robotic arms Patient side arm carts are labeled as C (camera), 1 (right), and 2 (left). Patient side arm carts with active and passive robotic arms are docked. A monopolar scissor is used in the right arm, and fenestrated bipolar instruments are used in the left arm. Image Credit: Authors

Colpotomy was performed by inserting the colpotomizer. The CO2 tubing was attached to the insufflation adaptor, and the abdomen was insufflated to 15 mmHg. The patient was positioned in the lithotomy position. The Veress insufflation was carried out through Palmer’s point (2 cm above and medial to the midclavicular line). The supraumbilical 12 mm camera port was inserted, followed by the placement of additional ports (Figure 4). A fenestrated bipolar grasper was placed in the left port, and either the monopolar scissors or the monopolar hook was placed in the right port. Figure 5 illustrates the docked patient side arm cart, with the middle camera arm, the right and left robotic arms, the monopolar scissors in the right arm, and the fenestrated bipolar instrument in the left arm.

The learning curve for robotic hysterectomy is 25 cases. During the training process, it is important to understand the parts and functioning of the robotic system. At least 10 cases are required to learn the docking process.

Preoperative preparation involved a thorough patient assessment, including a medical history review, lab tests, and imaging to evaluate the surgical risk. An anesthesia assessment was also conducted to evaluate potential risks and determine the anesthesia plan. Informed consent was obtained in detail, with a full discussion of the surgery’s benefits, risks, and alternatives explained to the patient. Antibiotic prophylaxis was administered to prevent infections, particularly surgical site infections, with a single dose of cephalosporin given 60 minutes before surgery. Alternatives may be used for patients with allergies. Pain management included the use of non-steroidal anti-inflammatory medications or acetaminophen postoperatively, while intraoperative management involved general anesthesia and opioids, such as fentanyl.

After prophylactic broad-spectrum antibiotic administration, all patients underwent robotic hysterectomy. Two robotic arms were found to be sufficient. An assistant accessory port was placed in the left upper quadrant to assist with suction/irrigation as needed in all the cases. One additional assistant port was placed in the right lower quadrant for traction with a myoma screw in three out of 10 cases. Following the port placement, the round ligaments were dissected first, followed by the infundibulopelvic ligaments. If the patient was under 50 years old, the utero-ovarian ligaments were dissected to preserve the ovaries. Fallopian tubes were removed in all the cases. Bilateral uterine arteries were sealed and dissected after skeletonization. After incising the vaginal cuff, hysterectomy tissues were removed through the vagina. Vaginal cuff closure was performed with a 2.0 barbed suture in one layer in a unidirectional manner. After completion of the surgery, the fascial defect was closed with delayed absorbable sutures, and the skin was closed with staplers.

Complications occurring intraoperatively or in the early postoperative period were recorded. Postoperative care was provided in the surgical intensive care unit for critical patients or patients with comorbidities. All patients were followed up in the gynecology inpatient service with routine postoperative care, including non-steroidal analgesics, antibiotics, and antiemetics.

Discharge criteria were based on the patient’s health status, including stable vitals, adequate pain control, and mobility. The VAS score was noted, and any complications, such as signs of bleeding, infection, or urinary retention, were monitored. The ability to void without issues was also assessed. Before discharge, follow-up care and instructions, including activity restrictions, were explained to the patient, and a follow-up appointment was scheduled.

## Results

Table 1 shows the mean age of surgery as 44.26 years, with an average BMI of 22.02 kg/m² (range 19-25 kg/m²) across all 15 cases. One patient among the cases was diagnosed with both hypertension and diabetes, while another was diagnosed with hypertension alone. The indications for surgery in the 15 cases were benign and included AUB (n=10, 83.33%), prophylactic robotic hysterectomy in a known case of breast malignancy (n=1, 8.33%), and carcinoma of the endometrium (n=1, 8.33%). Seven of the women had a history of abdominal surgery, including caesarean section and salpingectomy for sterilization.

**Table 1:** Clinical characteristics The mean age at surgery as 44.26 years, with an average BMI of 22.02 kg/m² (range: 19–25 kg/m²). The indications for surgery in the 15 cases were benign. They included AUB (n=10, 83.33%), prophylactic robotic hysterectomy in a known case of breast malignancy (n=1, 8.33%), and carcinoma of the endometrium (n=1, 8.33%). A uterine manipulator was used, specifically the Myoma screw. BS: bilateral salpingectomy, BSO: bilateral salpingo-ophorectomy, assistant port: 1, TH: total hysterectomy, LO: left ovary, RO: right ovary, AUB: abnormal uterine bleeding

Patient	Age, yr	BMI (kg/m^2^)	Number of prior abdominal surgeries	Indication	Pathology	Procedure performed
1	47	22.5	0	AUB	Adenomyosis	TH+ BS
2	50	23	1	AUB	Adenomyosis	TH+BS+LO
3	44	21	0	AUB	Leiomyoma	TH+BS
4	41	25	2	Risk reduction (k/c/o ca breast)	-	TH+BSO
5	39	20.4	1	AUB	Ovarian cyst	TH+BSO
6	44	23.1	0	AUB	Leiomyoma	TH+BS
7	44	22.6	2	AUB	Carcinoma endometrium	TH+BSO
8	48	21.8	0	AUB	Leiomyoma with endometrioma	TH+ BS+RO
9	46	24	1	AUB	Leiomyoma	TH+BS
10	40	20	0	AUB	Leiomyoma	TH+BS+LO
11	42	23	1	AUB	-	TH+BS
12	47	22	0	AUB	-	TH+BS+LO
13	45	23	0	Chronic pelvic pain with AUB	Leiomyoma	TH+BS
14	46	20	1	AUB	Adenomyosis	TH+BS
15	41	19	0	AUB	-	TH+BS

Table 2 shows the operative characteristics of the patient. The mean draping time was 8.53 minutes. The mean docking time was 9.46 minutes. The docking time was reduced from 25 minutes in the first case to five minutes in the 15th case. The mean operative time (from surgeon on console to surgeon off console) was three hours and three minutes (182.86 minutes).

**Table 2:** Operative characteristics The operative characteristics are displayed. The docking time was reduced from 25 minutes in the first case to five minutes in the 15th case. The mean operative time (from surgeon on console to surgeon off console) was three hours and three minutes (182.86 minutes).

Pt	Patient in time	Patient intubation time	Draping time (min)	Port placement	Docking time (min)	Surgeon on console	Surgeon off console
1	7.30 am	7.50 am	15	8.15 am	25	8.55 am	11.45 am
2	8.45 am	9 am	13	9.25 am	20	9.45 am	12.15 pm
3.	8.05 am	8.20 am	10	8.30 am	18	8.55 am	12.35 pm
4	9.05 am	9.20 am	9	9.30 am	10	10 am	12.35 pm
5	12.10 pm	12.40 pm	5	12.35 pm	5	12.50 pm	2.20 pm
6	9.30 am	9.45 am	10	10 am	5	10.30 am	2.30 pm
7	9.30 am	9.40 am	5	10.05 am	10	10.25 am	2 pm
8	8.45 am	9 am	6	9.30 am	12	10 am	4.30 pm
9	12.40 pm	12.50 pm	4	1.10 pm	5	1.35 pm	5 pm
10	9.30 am	10 am	15	10.30 am	5	10.40 am	2.30 pm
11	9 am	9.15 am	10	9.30 am	6	9.40 am	11.30 am
12	10 am	10.10 am	8	10.20 am	5	10.30 am	1.30 pm
13	9 am	9.10 am	7	9.20 am	5	9.30 am	11.45 am
14	10 am	10.10 am	6	10.20 am	6	10.30 am	1 pm
15	9.30 am	9.40 am	5	10 am	5	10.15 am	12.30 pm

Table 3 shows the operative characteristics. The average uterine weight was 101.33 g (range: 25-250 g). The average estimated blood loss was 120 mL (range: 30-400 mL). The mean operative time (from surgeon on console to surgeon off console) was three hours and three minutes (182.86 minutes). The mean VAS score for pain was 3.86. There was a drop in hemoglobin in four cases, with no postoperative blood transfusion required. The average length of hospital stay was 76.4 hours (three days and 4.4 hours). There were no significant intraoperative complications, except for right uterine artery bleeding. Omental adhesiolysis, sharp bladder dissection, myomectomy, ovarian cystectomy, and endometrioma excision were performed intraoperatively. No case was converted to open surgery.

**Table 3:** Operative characteristics The average uterine weight was 101.33 g (range: 25–250 g). The average estimated blood loss was 120 ml (range: 30–400 ml). The mean operative time (from surgeon on console to surgeon off console) was three hours and three minutes (182.86 minutes). The mean VAS score for pain was 3.86. The average length of hospital stay was 76.4 hours (three days and 4.4 hours). VAS: Visual Analogue Scale

SN	Estimated blood loss (ml)	Weight of excised uterus (g)	Operative time	Length of hospital stay (days)	Intraoperative complications	Intraop strategies	Conversion to open	VAS score	Drop in hemoglobin
										1	100	80	2 hr 40 m (160 m)	5	No	No	No	4	No
2	50	60	2 hr 30 m (150 m)	6	No	Omental adhesiolysis	No	3	No										
										3	80	100	3 hr 40 m (220 m)	6	No	No	No	4	No
4	30	25	2 hr 23 m (143 m)	5	No	Bladder dissected by sharp dissection	No	5	No										
										5	200	120	1 hr 30 m (90 m)	5	No	The right infundibulopelvic ligament densely adhered to the lateral pelvic wall. Adhesions removed by sharp dissection. Ovarian cyst removed	No	4	1 g%
6	150	250	4 hr (240 m)	5	No	Myomectomy	No	5	2 g%										
																				7	350	150	3 hr 25 m (205 m)	4	No	No	No	5	No
8	400	250	6 hr 30 m (390 m)	9	No	Right endometrioma excised, dense adhesions present in the pouch of Douglas. Sigmoid mobilization done	No	7	1 g%																				
										9	100	100	3 hr 25 m (205 m)	5	No	No	No	4	1 g%
10	90	120	3 hr 50 m (230 m)	5	Right uterine artery bleeding present, controlled with fenestrated bipolar	Left ovary cyst of 4 * 4 cm, cystectomy done	No	5	No										
										11	50	60	1 hr 50 m (110 m)	5	No	No	No	3	No
12	40	50	3 hr (180 m)	5	No	No	No	3	No										
										13	50	60	2 hr 15 m (135 m)	5	No	No	No	4	No
14	70	40	2 hr 30 m (150 m)	6	No	No	No	3	No										
										15	40	55	2 hr 15 m (135 m)	6	No	No	No	4	No

The hysterectomy audit at our medical college included patients with various conditions, such as uterine cancer, fibroids, endometriosis, uterine prolapse, chronic pelvic pain, abnormal bleeding (heavy bleeding not managed by other treatments), infections/abnormalities, and PID (chronic PID causing organ damage). The mean age of patients was 44.26 years, with a mean BMI of 22.02 kg/m². One patient had both hypertension and diabetes, while another had hypertension. The indications for surgery were benign, including AUB (83.33%), prophylactic hysterectomy for breast cancer (8.33%), and endometrial cancer. Seven patients had a history of abdominal surgery.

The youngest patient undergoing robotic hysterectomy and cystectomy was a 39-year-old with a 10 × 10 cm ovarian cyst and four years of AUB. Despite hormonal treatment at multiple hospitals, her symptoms persisted. Living in a remote area with a complete family and impaired quality of life, the decision was made to proceed with surgery due to challenges in follow-up.

One ovary was preserved in four patients, and the other, which appeared necrotic, was removed intraoperatively; its histopathology report was benign. One patient had endometrial carcinoma, another had a history of breast cancer, and the third had a 10 × 10 cm ovarian cyst with difficulty monitoring ovarian tumor markers and a family history of breast cancer. Given these factors, the ovaries were removed.

The mean operative time was 182.86 minutes, with docking time decreasing from 25 to 5 minutes by the 15th case. The mean hospital stay was 5.4 days, with most patients being discharged between days 5 and 7. Seven patients had an uneventful recovery, while others experienced mild issues such as abdominal pain, epigastric pain, or vaginal discharge, all of which resolved with treatment. Intraoperative complications included uterine artery bleeding, bladder adhesions from prior cesarean sections, and dense adhesions in various areas, all of which were managed appropriately. Myomectomy and ovarian cyst excision were performed in specific cases. The average uterine weight was 101.33 g, and the average blood loss was 120 mL. Pain was minimal (mean VAS score: 3.86), with a small decrease in hemoglobin in four cases, though no transfusions were needed. There were no emergency room visits or delayed wound healing, and only one readmission for upper abdominal pain. Histopathology revealed benign findings in 14 out of 15 cases.

## Discussion

The most common surgery performed in gynecologic practice is hysterectomy. Hysterectomies are most commonly performed for benign conditions such as fibroids, endometriosis, adenomyosis, and prolapse. Vaginal hysterectomy should be performed whenever feasible, according to American College of Obstetricians and Gynecologists. When it is not feasible, the surgeon should follow different surgical approaches [6]. Laparoscopic hysterectomy has a steep learning curve and requires advanced training and skills, which limit its adoptability. Robotic minimally invasive surgery offers wrist-like motion for better precision, mobility, dexterity, and 3D vision, which enhances the view of the operative field. This case series demonstrates the efficient use of the robotic minimally invasive approach for both benign and malignant cases, especially in cases involving a bulky uterus with multiple fibroids.

The purpose of introducing robotic surgery at our institution was to improve the performance of complex cases, such as endometriosis and oncological procedures, safely via a minimally invasive approach, with a shorter learning curve compared to laparoscopy. There was a significant reduction in the percentage of abdominal hysterectomies, especially in complex cases with a bulky uterus and prior abdominal surgeries [6].

In the present study, blood loss was significantly less, with no blood transfusion required in the majority of cases. A drop in hemoglobin was observed in four cases, but none required a blood transfusion in the postoperative period. Patients experienced satisfactory recovery.

Various studies have highlighted the advantages of minimally invasive hysterectomy, including reduced bleeding, lower perioperative and postoperative complication rates, and shorter hospital stays [7]. The longer operation time is primarily due to the docking procedure, where the robotic arms are fixed to the ports. However, with experience, the docking time can be reduced [7]. In our study, the docking time significantly decreased from 25 minutes for the first case to five minutes for the 15th case.

Studies have shown that robotic hysterectomy offers more advantages than laparoscopy, especially for patients with large uteri or obesity [8]. This indigenous robotic system was very surgeon-friendly, with minimal blood loss. It featured an open console that allowed other surgeons to observe the surgical steps, a 3D camera system, and articulating robotic arms with 7° of freedom for smooth, tremor-free movement and precise suturing with minimal learning curve.

Research indicates that performing 40-50 robotic cases is sufficient to achieve a level of expertise in robotic surgeries. The main advantage of robotic surgery lies in the ease of operation, with the surgeon sitting comfortably at the open console and using a self-maneuvering camera system. One meta-analysis demonstrated that robotic cases had a shorter hospital stay compared to laparoscopic surgeries. However, no significant advantages were found for robotic surgery over laparoscopy regarding clinical outcomes in hysterectomy for benign disease [9].

The complication rates were similar between the two approaches. Intraoperative strategies such as omental adhesiolysis, sigmoid colon mobilization, endometrioma excision, and release of dense adhesions can be efficiently and safely performed using robotic surgery. A hospital-based study showed that robotic surgery can be performed more rapidly than conventional laparoscopic surgery once the surgeon becomes proficient, after approximately 75 surgeries [10].

In a study by Okumura et al., retrospective evaluation of surgical outcomes, estimated blood loss, and the weight of excised uteri were examined. The study found that, regardless of the surgeon's experience, the net operative time was shorter in the robotic hysterectomy group compared to the total laparoscopic hysterectomy (TLH) group. Additionally, estimated blood loss was significantly lower in the robotic hysterectomy group compared to the TLH group [11].

Azadi et al. observed that the robotic approach provided benefits over TLH in patients with high BMI. They demonstrated the safe and efficient use of the robotic approach in both benign and malignant cases. The rate of complications, such as conversion to laparotomy, urinary tract injury, and vaginal cuff dehiscence, was higher in the laparoscopic approach [12].

Herrinton et al. found that for both complex and non-complex patients, robotic and conventional surgeries were similar in terms of average length of stay, risks of readmission within 90 days, reoperation, and most complications. However, the VAS score for pain was significantly lower in the robotic surgery group, with better postoperative recovery and improved quality of life [10].

Tabrizi et al., in a systematic review, noted that minimally invasive surgeries may offer comparable survival outcomes to open surgery but carry a higher risk of recurrence, especially with laparoscopy. Some studies in the review observed that minimally invasive surgeries had longer operative times but resulted in less blood loss compared to open procedures [13].

Dai et al. found that the robotic radical hysterectomy group had a longer operative time, shorter hospital stays, more dissected lymph nodes, and less blood loss compared to the laparoscopic radical hysterectomy group [14].

However, robotic surgery has some disadvantages, including longer operative times and higher costs. The draping time for robotic arms requires practice from the nursing staff, but training can significantly reduce this time. In our study, draping time decreased from 15 minutes in the first case to eight minutes. The cost of robotic hysterectomy is a major disadvantage, as it is typically 1.5 to three times higher than laparoscopic hysterectomy. The additional costs, such as maintenance, instrument, and drape costs, contribute to this higher price. However, as the frequency of use increases, the manufacturing costs of robots and equipment are expected to decrease.

The robotic system components require a large operating theatre. Four robotic arms must be parked in a safe location. The surgeon's console, patient cart, and endoscopic tower should be properly organized and managed by trained staff and surgeons. The size of the ports is also larger compared to laparoscopic ports, and the number of ports is typically greater. Cosmetic outcomes may be compromised. The heavy robotic arms may sometimes make contact with the patient's body parts, such as the head or thorax. The bedside team surgeons may experience ergonomic difficulties when retracting with the myoma screw or suctioning, and may occasionally be injured by the robotic arm. The gentle, minimal, and fine wrist movements the system requires necessitate a learning curve, which can be minimized as the number of cases performed increases.

Despite these disadvantages, several studies have shown that robotic hysterectomy is preferable in almost all gynecological cases, including those involving leiomyoma, endometriosis, and oncological surgeries.

## Conclusions

Robotic hysterectomy is the preferred surgical procedure due to better ergonomics and a shorter learning curve, especially in cases involving bulky uteri and endometriosis, where the seven degrees of freedom of the robotic arms can aid in easier dissection. In the future, the reduced cost of indigenous robotic systems will make them available in tertiary care units for performing complex gynecologic procedures, such as those for endometriosis, bulky uteri with large fibroids, and oncological cases.
